# Purinergic P2X_3_ receptors in the carotid body as new therapeutic targets for controlling heart failure

**DOI:** 10.1007/s11302-023-09945-y

**Published:** 2023-05-22

**Authors:** Ximeng He, Zhichao Zhou

**Affiliations:** 1 School of Acupuncture and Tuina/International Joint Research Centre On Purinergic Signalling, Chengdu University of Traditional Chinese Medicine, 37 Shi-Er-Qiao Road, Chengdu, 610075 China; 2 Acupuncture & Chronobiology Key Laboratory of Sichuan Province, Chengdu, 610075 China; 3 Division of Cardiology, Department of Medicine Solna, Karolinska University Hospital, Karolinska Institutet, 17176 Stockholm, Sweden

**Keywords:** Purinergic P2X_3_ receptors, Carotid body, Heart failure

## Abstract

Heart failure is associated with multiple mechanisms, including sympatho-excitation, and is one of the leading causes of death worldwide. Enhanced carotid body chemoreflex function is strongly related to excessive sympathetic nerve activity and sleep-disordered breathing in heart failure. How to reduce the excitability of the carotid body is still scientifically challenging. Both clinical and experimental evidence have suggested that targeting purinergic receptors is of great potential to combat heart failure. In a recent study, Lataro et al. (Lataro et al. in Nat Commun 14:1725, 5) demonstrated that targeting purinergic P2X_3_ receptors in the carotid body attenuates the progression of heart failure. Using a series of molecular, biochemical, and functional assays, the authors observed that the carotid body generates spontaneous, episodic burst discharges coincident with the onset of disordered breathing in male rats with heart failure, which was generated by ligating the left anterior descending coronary artery. Moreover, P2X_3_ receptor expression was found to be upregulated in the petrosal ganglion chemoreceptive neurons of rats with heart failure. Of particular note, treatment with a P2X_3_ antagonist rescued pathological breathing disturbances, abolished episodic discharges, reinstated autonomic balance, attenuated cardiac dysfunction, and reduced the immune cell response and plasma cytokine levels in those rats.

## Commentary

Heart failure has been associated with sympathetic excitation and parasympathetic withdrawal, increased sympathetic-respiratory coupling, and irregular breathing patterns. These alterations have been attributed to an enhanced carotid body chemoreceptor drive [1] and are strongly related to a worsening prognosis. However, bilateral resection of the carotid bodies in humans may result in clinical exacerbation of nocturnal hypoxaemia and permanent elimination of the hypoxic ventilatory responses in normocapnic conditions [1, 2]. Hence, there is an urgent need to develop novel therapeutic strategies to normalize the excitability of carotid body. ATP acts as a co-transmitter for many types of neurons that provide neural control of the heart and plays an important role in maintaining the normal vascular tone and cardiac reflex activity via activation of purinergic receptors [3]. Of note, the pathological potential of abnormal purinergic signaling that contributes to and aggravates various types of cardiovascular diseases, including heart failure, has been increasingly recognized. Despite targeting purinergic receptors mainly with a focus on cardiomyocytes having been shown to have promising outcomes [4], the effect of purinergic receptor modulation in the carotid body on heart failure remains to be determined. In a recent paper published in Nature Communications by Lataro et al. [5], the authors identify purinergic P2X_3_ receptors in the carotid body as an important therapeutic target for the treatment of heart failure in male rats.

Both purinergic P1 and P2 receptors are expressed and involved in excitability of the carotid body [6, 7]. Previous work from the same group demonstrated that P2X_3_ receptors are upregulated in the carotid body and that this is causal for the etiology of hyperreflexia of chemoreceptive petrosal neurons and hyperactivity of the sympathetic nervous system in spontaneously hypertensive rats [8]. In the present study, the authors continued to focus on P2X_3_ receptors and observed that they are upregulated in the petrosal chemoreceptive neurons from rats with heart failure [5], while the expression of other receptors, e.g., P2X_2_ receptors, that could form a heterodimeric receptor with P2X_3_ receptor, is not altered in heart failure [9]. Although the authors provided evidence of the P2X_3_ receptor protein expression in heart failure tissue, the P2X_3_ receptor expression comparison between healthy and heart failure tissue was mainly performed by measuring the mRNA levels where the housekeeping gene β-actin was used. There is evidence showing cross-talk between actin function and ATP-mediated P2X signaling [10, 11], so using additional housekeeping genes would be of value to support the altered P2X_3_ expression. In addition to expression, the sensitivity of the purinergic receptors may reflect a better functional measure of the receptors [12, 13].

The authors applied a pharmacological P2X_3_ receptor antagonist to further investigate the function of P2X_3_ receptors and their therapeutic impact on heart failure [5]. They found that focal delivery of a P2X_3_ receptor antagonist (AF-353; 15 nl, 20 mM) to the bilateral carotid bodies of rats with heart failure improved and restored autonomic balance and normalized chemoreflex hyperreflexia in heart failure rats. Both the hyperreflexia of thoracic sympathetic activity and abdominal nerve chemoreflex-evoked expiratory discharge were normalized by P2X_3_ receptor blockade in the carotid bodies and most thoracic sympathetic activity was positively modulated in the expiratory phase. These data provide the insights that P2X_3_ receptors within the carotid body are mostly responsible for the hyperreflexia of sympathetic and expiratory chemoreflex responses in heart failure rats. Given the unclear role of P2X_2_ receptors in heart failure and possible presence of P2X_2_/X_3_ heterodimeric receptors [6], it is likely that a high concentration of P2X_3_ receptor antagonist AF-353 applied in the in situ preparation may also act at P2X_2_ receptors and result in non-specific effects, which could partially contribute to the therapeutic effect. Genetic tools, such as P2X_3_ receptor knockout, could be considered to further support the findings.

The study further reveals that systemic blockade of the P2X_3_ receptor increases respiratory stability and attenuates heart failure progression. The authors applied the antagonist, AF-130, which does not cross the blood–brain barrier and so excludes central P2X_3_ antagonism and found that AF-130 eliminated abnormal carotid sinus nerve discharges and subsequent respiratory disturbances in rats with heart failure. Carotid sinus nerve discharge is often induced by hypoxia or hypercapnia to elicit reflex ventilatory and restore arterial blood oxygen tension [1]. Interestingly, the team revealed that under normal blood gas saturation, the carotid sinus nerve of heart failure rats generated spontaneous discharges concomitant with increased expiratory motor activity. ATP is the major excitatory transmitter released by the glomus cells of the carotid body to activate sensory endings of the sinus nerve afferent fibers. The authors suggested that the spontaneous episodic discharges are caused by an intervallic release of ATP, which acts on P2X_3_ receptors to selectively drive activation of pathways and promote aberrant expiratory activity to trigger inspiratory instability. The exact pathological mechanism that drives ATP release remains to be elucidated. However, measurement of ATP levels or promoting metabolism of ATP in the rat model could be considered to provide additional mechanistic insights. Of further importance, P2X_3_ receptor antagonism also ameliorates cardiac dysfunction and inflammation in heart failure rats, as chronic blockade of P2X_3_ receptors improved cardiac output, lowered heart and lung weights, and reduced heart failure biomarkers and systemic inflammation. These data provide solid experimental evidence for the treatment of heart failure by targeting the P2X_3_ receptor.

Overall, this study provides new insights on therapeutic strategy for the treatment of heart failure by targeting purinergic signaling. Pharmacological inhibition of purinergic P2X_3_ receptors in the carotid body of rats with heart failure can bring a series of beneficial effects. This includes rescuing pathological breathing disturbances, abolishing episodic discharges, reinstating autonomic balance, and attenuating cardiac dysfunction [5]. As the expression and distribution of purinergic receptors varies from species to species [3], challenges for the future translational study will be the identification of the P2X_3_ receptor expression and function in the human carotid body under the pathological condition of heart failure. Existing evidence has shown that P2X_3_ receptor expression is not detected by microarray and qPCR in carotid bodies removed unilaterally from patients undergoing radical neck dissection [7]. More studies are needed to investigate the expression and function of this receptor in humans with heart failure. Also, only male rats were used in the present study; therefore, an additional focus may be put on female patients, as the ATP-mediated P2X_3_ receptor signaling could be different between males and females [14]. Of note, the P2X_3_ receptor antagonist AF-130 used in the present study has gone through phase 1 clinical trial. Better rational design and development of P2X_3_ antagonists are expected to lead to the introduction of a successful clinical drug in the future.

## Data Availability

No data was generated for this article.
